# Recognition of Emotion by Brain Connectivity and Eye Movement

**DOI:** 10.3390/s22186736

**Published:** 2022-09-06

**Authors:** Jing Zhang, Sung Park, Ayoung Cho, Mincheol Whang

**Affiliations:** 1 Department of Emotion Engineering, Sangmyung University, Seoul 03016, Korea; 2 Department of Human-Centered Artificial Intelligence, Sangmyung University, Seoul 03016, Korea

**Keywords:** emotion recognition, attention, eye movement, brain connectivity

## Abstract

Simultaneous activation of brain regions (i.e., brain connection features) is an essential mechanism of brain activity in emotion recognition of visual content. The occipital cortex of the brain is involved in visual processing, but the frontal lobe processes cranial nerve signals to control higher emotions. However, recognition of emotion in visual content merits the analysis of eye movement features, because the pupils, iris, and other eye structures are connected to the nerves of the brain. We hypothesized that when viewing video content, the activation features of brain connections are significantly related to eye movement characteristics. We investigated the relationship between brain connectivity (strength and directionality) and eye movement features (left and right pupils, saccades, and fixations) when 47 participants viewed an emotion-eliciting video on a two-dimensional emotion model (valence and arousal). We found that the connectivity eigenvalues of the long-distance prefrontal lobe, temporal lobe, parietal lobe, and center are related to cognitive activity involving high valance. In addition, saccade movement was correlated with long-distance occipital-frontal connectivity. Finally, short-distance connectivity results showed emotional fluctuations caused by unconscious stimulation.

## 1. Introduction

Studies have shown that different brain regions participate in various perceptual and cognitive processes. For example, the frontal lobe is related to thinking and consciousness, whereas the temporal lobe is associated with processing complex stimulus information, such as faces, scenes, smells, and sounds. The parietal lobe integrates a variety of sensory inputs and the operational control of objects, while the occipital lobe is related to vision [1].

The brain is an extensive network of neurons. Brain connectivity refers to the synchronous activity of neurons in different regions and may provide useful information on neural activity [2]. Mauss and Robinson [3] suggested that emotion processing occurs in distributed circuits, rather than in specific isolated brain regions. Analysis of the simultaneous activation of brain regions is a robust pattern-based analysis method for emotional recognition [4]. Researchers have developed methods to capture asymmetric brain activity patterns that are important for emotion recognition [5].

Users search massive amounts of information until they find something useful [6]. However, although the information is presented visually, users do not recognize it, because of a lack of attention. The cortical area known as the frontal eye field (FEF) plays a vital role in the control of visual attention and eye movements [7].

Eye tracking is the process of measuring eye movements. Eye tracking signals imply the user’s subconscious behaviors and provide essential clues to the context of the subject’s current activity [8], which allow us to determine what elicits users’ attention.

The brain activity is significantly related to eye movement features involving pupil, saccade, and fixation. Our pupils change their size accordingly [9] when one is stimulated from resting to emotional states. The saccade is a decision made every time we move our eyes [10,11]. Decisions are influenced by one’s expectations, goals, personalities, memories, and intentions [12].

A gaze is a potent social cue. For example, mutual gaze often implies threat or evasion, signaling submission or avoidance [13,14,15,16]. Eye gaze processing is one of the bases for social interactions, because the neural substrate for gaze processing is an essential step in developing neuroscience for social cognition [17,18].

By analyzing eye movement data, such as gaze position and gaze time, researchers can obtain explanations for multiple cognitive operations involving multiple behaviors [19]. For example, language researchers can use eye-tracking to analyze how people read and understand spoken language. Consumer researchers can study how shoppers make purchases. Researchers can gain a better cognitive understanding by integrating eye tracking with neuroimaging technologies (e.g., fMRI and EEG) [20]. 

Table 1 compares the few studies on eye movement features and EEG signals with an interest in producing a robust emotion-recognition model [21]. Wu et al. [22] integrated functional features from EEG and eye movements with deep canonical correlation analysis (DCCA). Their classification achieved 95.08% ± 6.42% accuracy on SEED public emotion EEG datasets [23]. Zheng et al. [24] used a multimodal depth neural network to incorporate eye movement and EEG signals to improve recognition performance. The results demonstrated that modality fusion with deep neural networks significantly enhances the performance compared with a single modality. Soleymani [25] learned that the decision-level fusion strategy is more adaptive than feature-level fusion when incorporating EEG signals and eye movement data. They also found that user-independent emotion recognition can perform better than individual self-reports for arousal assessment. While studies focused on improving recognition accuracy, currently, there is a lack of understanding of the relationship between brainwave connectivity and eye movement features (fixation, saccade, and left and right pupils). Specifically, we do not know how the functional relationship varies according to visual content’s emotional characteristics (valence, arousal).

In this study, our research question involves the functional characteristics of brainwave connectivity and eye movement eigenvalues in valence-arousal emotions in a two-dimensional emotional model. We hypothesized that when viewing video content, the activation features of brain connections are significantly related to eye movement characteristics. We divided and analyzed brainwave connectivity into three groups: (1) long-distance occipital-frontal connectivity, (2) long-distance prefrontal and temporal, parietal, and central connectivity, and (3) short-distance connectivity, including frontal-temporal, frontal-central, temporal-parietal, and parietal-central connectivity. We applied k-means clustering to distinguish emotional feature responses, and eye movement eigenvalues were further differentiated. We then analyzed the relationship between eye movements and brain wave connectivity, depicting the differential characteristics of a two-dimensional emotional model. 

## 2. Materials and Methods

We adopted Russell’s two-dimensional model [26], where emotional states can be defined at any valence or arousal level. We invited participants to view emotion-eliciting videos with varying valences (i.e., from unpleasant to pleasant) and arousal levels (i.e., from relaxed to aroused). To understand brain connectivity and causality of brain regions according to different emotions, we used supervised learning to classify emotional and non-emotional states, and extract eye movement feature values associated with such different emotional states to analyze the relationship between brain activity and eye movement.

### 2.1. Stimuli Selection

**Figure 1:**
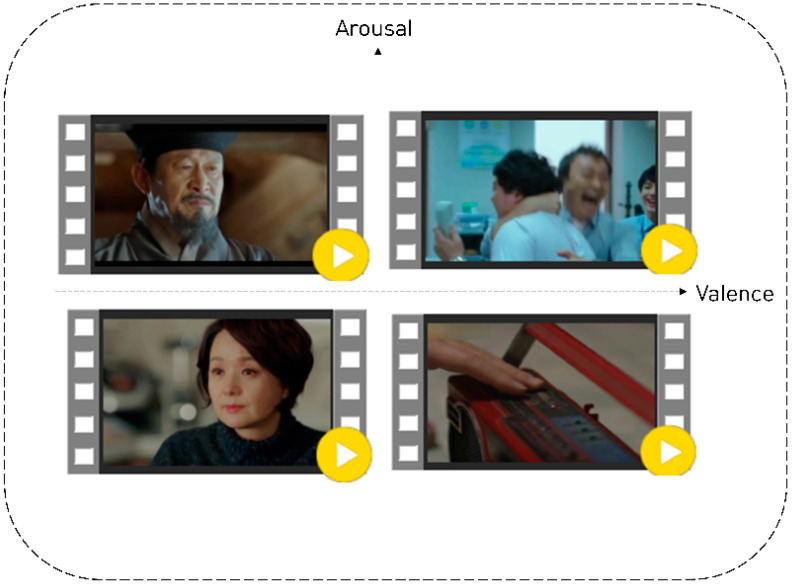
Video stimulus for each quadrant on a two-dimensional model.

We edited 6-min video clips (e.g., dramas or films) to elicit emotions from the participants. The content used to induce emotional conditions (valence and arousal) was collected in a two-dimensional model. To ensure that the emotional videos were effective, we conducted a stimulus selection experiment prior to the main experiment. We selected 20 edited dramas or movies containing emotions; five video clips were used for each quadrant in the two-dimensional model. Thirty participants viewed the emotional videos and responded to a subjective questionnaire. They received USD 20 for their participation in the study. Among the five video clips, the most representative video for each of the four quadrants in the two-dimensional model was selected (see Figure 1). Four stimuli were selected for the main experiment.

### 2.2. Experiment Design

The main experiment had a factorial design of two (valence: pleasant and unpleasant) × two (arousal: aroused and relaxed) independent variables. The dependent variables included participants’ brainwaves, eye movements (fixation, saccade, and left and right pupils), and subjective responses to a questionnaire.

### 2.3. Participants

We conducted an a priori power analysis using the program G*Power with the power set at 0.8 and α = 0.05, d = 0.6 (independent *t*-test), two-tailed. These results suggest that an N value of approximately 46 is required to achieve appropriate statistical power. Therefore, 47 university students were recruited for the study. Participants’ ages ranged from 20 to 30 years (mean = 28, STD = 2.9), with 20 (44%) men and 27 (56%) women. We selected participants with a corrective vision ≥ 0.8, without any vision deficiency, to ensure reliable recognition of visual stimuli. We recommended that the participants sleep sufficiently and refrain from smoking and consuming alcohol and caffeine the day before the experiment. As the experiment required valid recognition of the participant’s facial expression, we limited the use of glasses and cosmetic makeup. All participants were briefed on the purpose and procedure of the experiment, and signed consent was obtained from them. They were then compensated for their participation by payment of a fee.

### 2.4. Experimental Protocol

**Figure 2:**
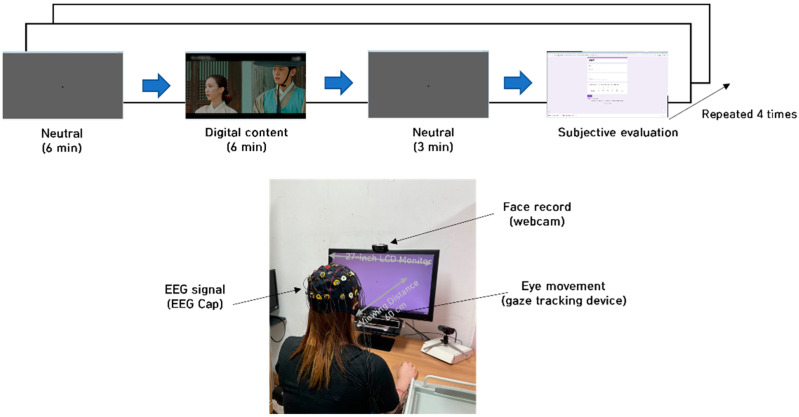
Experimental protocol and configuration.

Figure 2 outlines the experimental process and the environment used in this study. The participants were asked to sit 1 m away from a 27-inch LCD monitor. A webcam was installed on the monitor. Participants’ brainwaves (EEG cap 18 Ch) and eye movements (gaze tracking device) were acquired, in addition to subjective responses to a questionnaire. We set the frame rate of the gaze-tracking device to 60 frames per second. Participants viewed four emotion-eliciting videos and responded to a questionnaire after each viewing session.

## 3. Analysis

**Figure 3:**
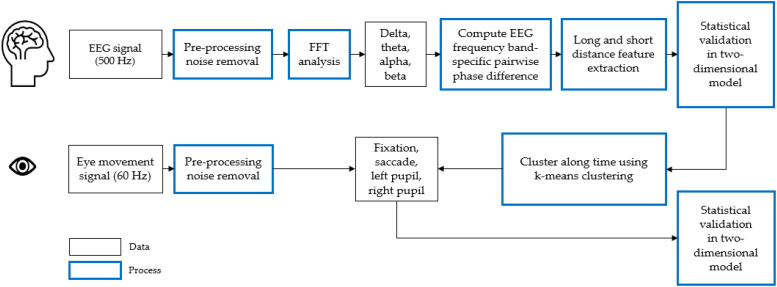
The process of brain connectivity analysis.

Our brain connectivity analysis methods were based on Jamal et al. [27], as outlined in Figure 3. The process consisted of seven stages: (1) sampled EEG signals at 500 Hz, (2) removed the noise through pre-processing, (3) conducted fast Fourier transform (FFT) at 0–30 Hz, (4) conducted band pass filter with delta (0 Hz–4 Hz), theta (4 Hz–8 Hz), alpha (8 Hz–12 Hz), and beta (12 Hz–30 Hz), (5) processed continuous wavelet transform (CWT) with complex Morlet wavelet, (6) computed the EEG frequency band-specific pairwise phase difference, and (7) determined the optimal number of states in the data using incremental k-means clustering.

**Figure 4:**
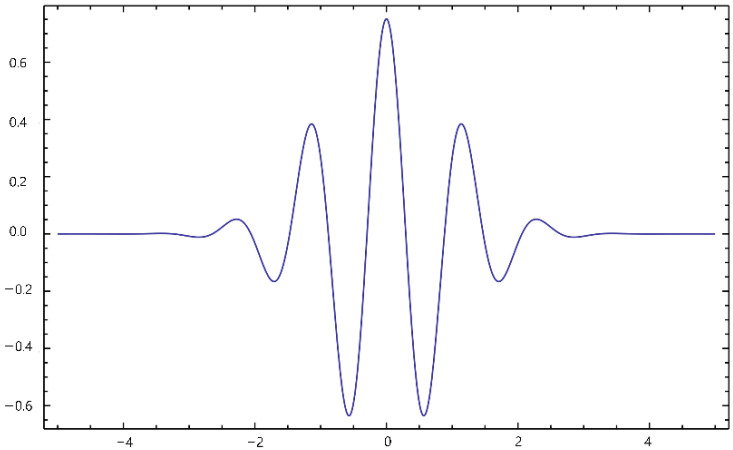
The Morlet wavelet graph.

We used the CWT with a complex Morlet wavelet as the basis function to analyze the transient dynamics of phase synchronization. In contrast to the discrete Fourier transform (DFT), it has a short vibration signal and an expiration date for the vibration wave. Figure 4 shows the Morlet wavelet graph. The CWT operates with a signal with scaled and shifted versions of a basic wavelet.

Therefore, it can be expressed as the formula below in Equation (1), where a is a scale factor and b is a shift factor. Being continuous, infinite wavelets can be shifted and scaled:

(1) $$X_{w}\left(a,b\right)=\frac{1}{{\left|a\right|}^{\frac{1}{2}}}\;\int_{-\infty }^{\infty }x\left(t\right)\bar{\varphi }\left(\frac{t-b}{a}\right)dt$$

## 4. Results

We will present the results of the participants’ subjective evaluation and brain connectivity analysis, followed by the results of eye movement analysis.

### 4.1. Subject Evaluation

We compared the subjective arousal and valence scores between the four emotion-eliciting conditions (pleasant-aroused, pleasant-relaxed, unpleasant-relaxed, and unpleasant-aroused). We conducted a series of ANOVA tests on the arousal and valence scores. Post-hoc analyses using Tukey’s HSD were conducted by adjusting the alpha level to 0.0125 per test (0.05/4).

**Figure 5:**
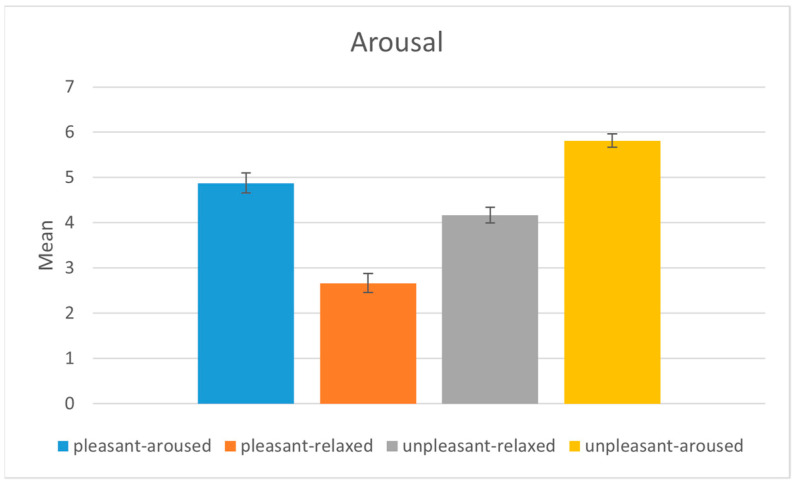
Analysis of the arousal values between the four emotion-eliciting conditions.

The mean arousal scores were significantly higher in the aroused conditions (pleasant-aroused, unpleasant-aroused) than in the relaxed conditions (pleasant-relaxed, unpleasant-relaxed) (*p* < 0.001), as shown in Figure 5. The pairwise comparison of the mean arousal scores indicated that the scores were significantly different from one another, as shown in Table 2. The results indicate that participants reported congruent emotional arousal with the target emotion of the stimulus.

**Figure 6:**
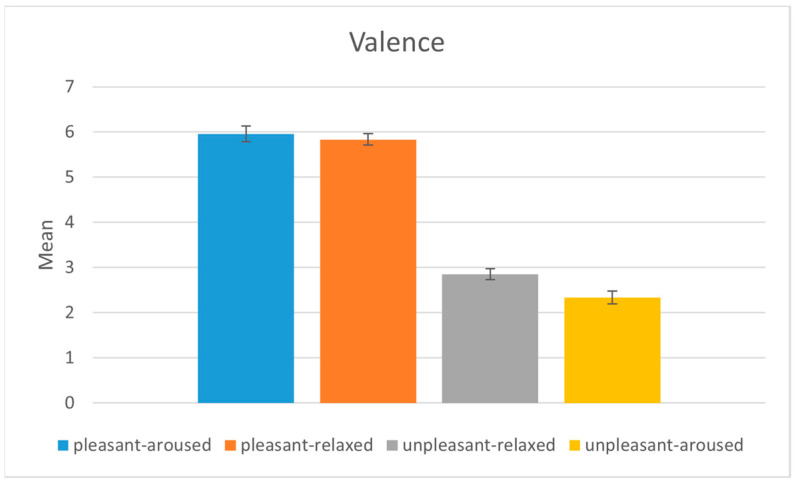
Analysis of the valence values between the four emotion-eliciting conditions.

The results indicated that the mean valence scores were significantly higher in the pleasant conditions (pleasant-aroused, pleasant-relaxed) than in the unpleasant conditions (unpleasant-aroused, unpleasant-relaxed), *p* < 0.001, as shown in Figure 6. The pairwise comparison of the mean valence scores indicated that the scores were significantly different from one another, except for two comparisons, as shown in Table 3. The results indicate that participants reported congruent emotional valence with the target emotion of the stimulus.

### 4.2. Brain Connectivity Features

**Figure 7:**
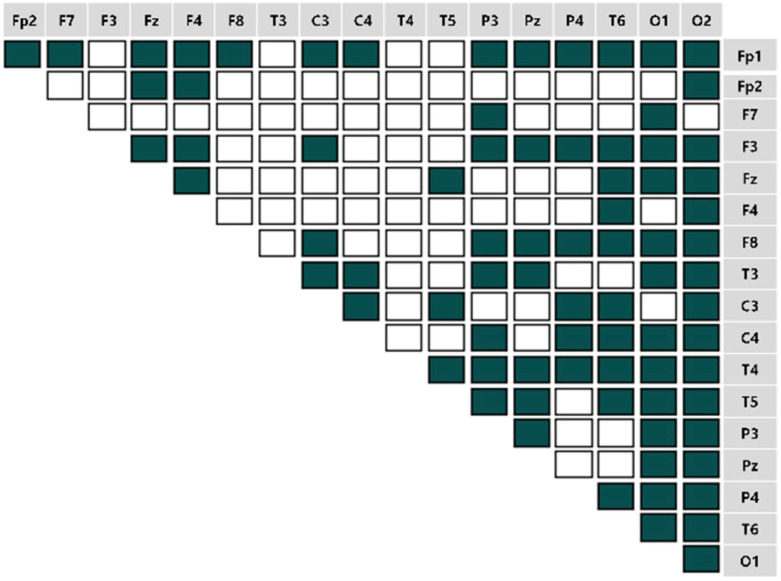
The brain connectivity map in the pleasant-aroused condition.

**Figure 8:**
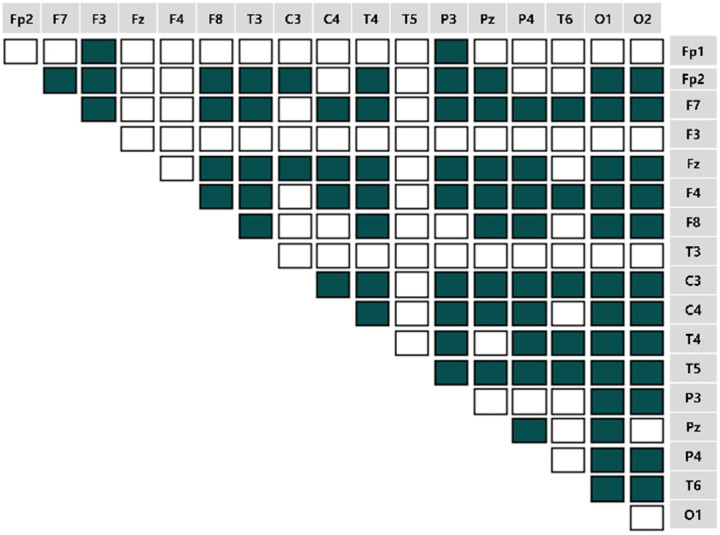
The brain connectivity map in the pleasant-relaxed condition.

**Figure 9:**
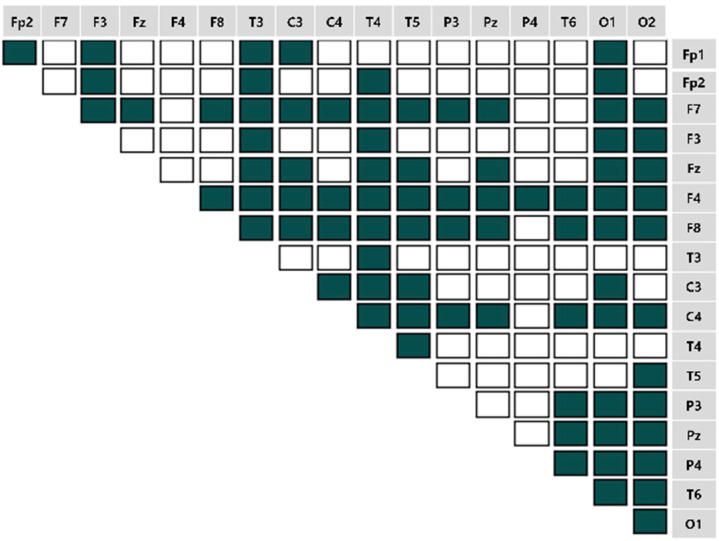
The brain connectivity map in the unpleasant-relaxed condition.

**Figure 10:**
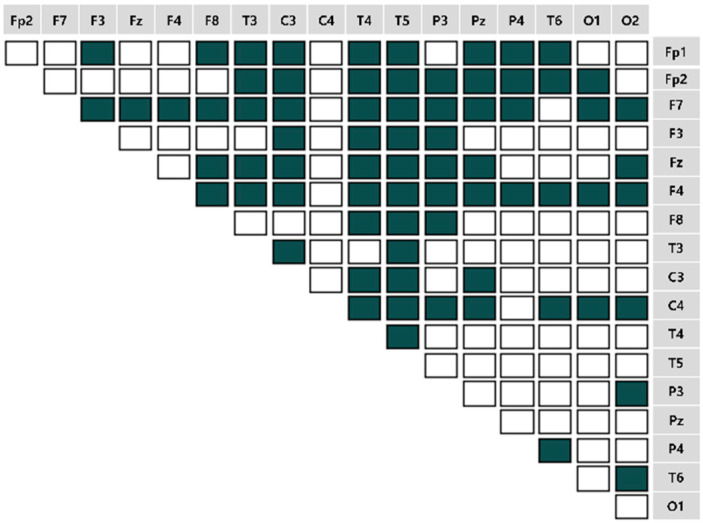
The brain connectivity map in the unpleasant-aroused condition.

We computed the EEG frequency band-specific pairwise phase differences for each emotion-eliciting condition, as shown in Figure 7, Figure 8, Figure 9 and Figure 10. A total of 153 pairwise features were analyzed. If the power differences between the two brain regions are lower than the mean power value, the connectivity is relatively strong. Such cases were marked as unfilled (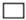). 

**Figure 11:**
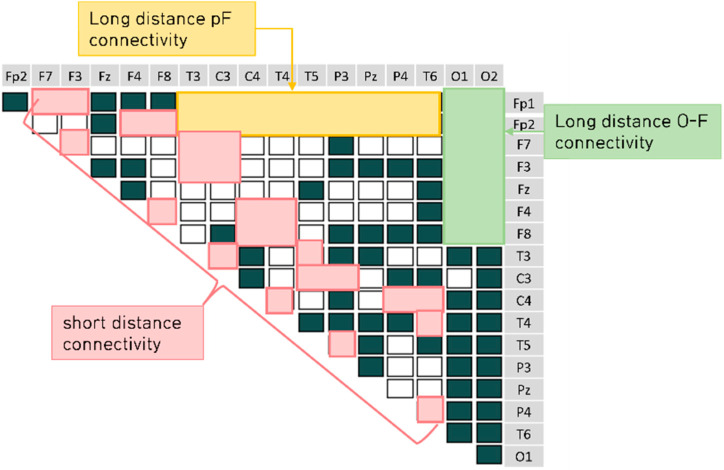
The three distance connectivity groups in the brain connectivity map.

We further analyzed the long- and short-distance connectivity of the extracted features. The connectivity of the frontal and occipital lobes can predict the process of information transmission to the occipital lobe after emotion is generated (marked in green in Figure 11). The eigenvalue was the average (N = 47) of the connectivity sum of the two channels defined by the long-distance O-F connectivity.

The prefrontal cortex is involved in emotion regulation, recognition, judgment, and reasoning. The connectivity of the prefrontal lobe to the temporal lobe, parietal lobe, and center helps to understand the information processing process of visual-emotional stimuli (marked in yellow in Figure 11). The eigenvalue was the average (N = 47) of the connectivity sum of the two channels defined by the long-distance prefrontal connectivity.

Long- and short-range connectivity features have been extensively studied for their ability to process social emotions and interactions. Short-distance connectivity characteristics can determine the brain’s different states during negative emotions, especially those related to the central-parietal lobe connectivity. We considered a distance of less than 10 cm as short connectivity (marked pink in Figure 11). The eigenvalue was the average (N = 47) of the connectivity sum of the two channels defined by the short-distance connectivity.

#### 4.2.1. Characteristics of Three Distance Connectivity

**Figure 12:**
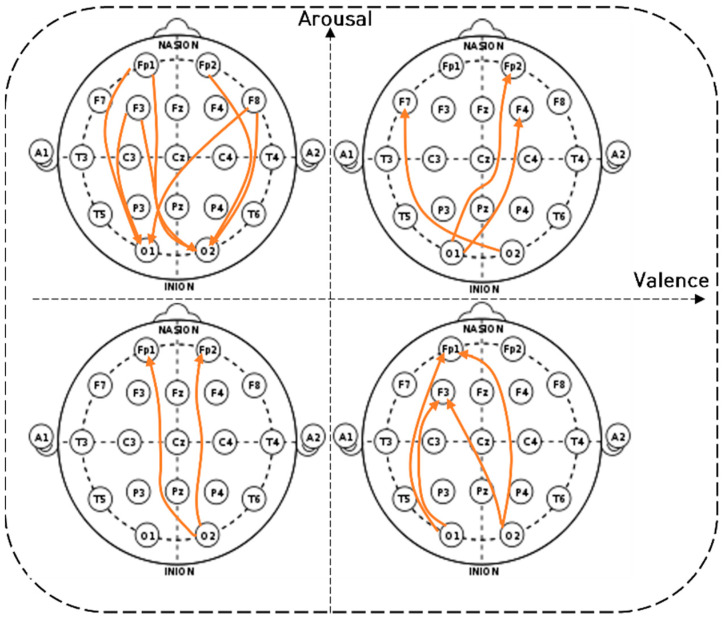
The long-distance connectivity of the occipital and frontal lobes (LD_O-F connectivity) of the beta wave.

Figure 12 depicts the long-distance connectivity of the occipital and frontal lobes (LD_O-F connectivity) of the beta wave in the visual comparison diagram of the two-dimensional model. O-F connectivity in the unpleasant-aroused condition had the strongest connectivity. In the pleasant-relaxed condition, bi-directional connectivity was observed between the left frontal and occipital lobes. In the unpleasant-relaxed condition, bidirectional connectivity was observed from the right occipital to the frontal lobe. In the pleasant-aroused condition, cross-hemispheric connectivity was observed between the frontal and occipital lobes.

**Figure 13:**
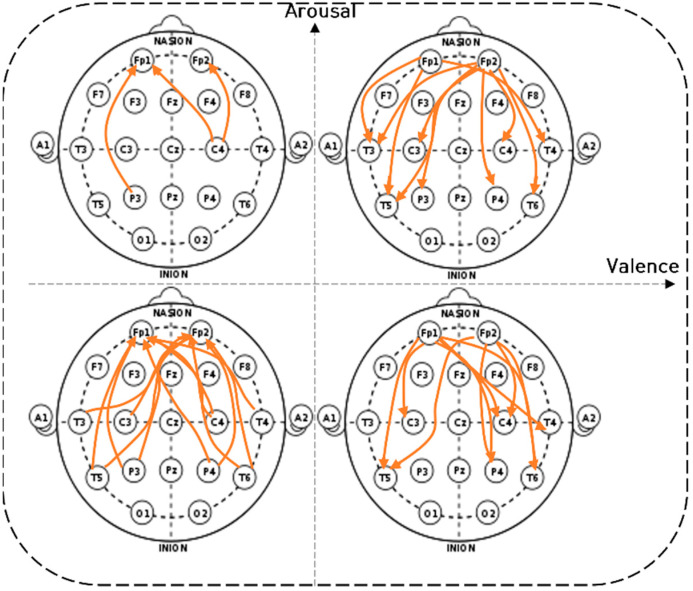
The long-distance connectivity of the prefrontal and temporal lobes, parietal lobes, and central (LD_pF connectivity) of the beta wave.

Figure 13 depicts the long-distance connectivity of the prefrontal and temporal lobes, parietal lobes, and central (LD_pF connectivity) beta waves in the visual comparison diagram of the two-dimensional model. In pleasant-aroused and unpleasant-relaxed conditions, the right prefrontal lobe was strongly connected to the central, parietal, and temporal lobes of both hemispheres. In the pleasant-relaxed condition, there was strong connectivity in the left prefrontal–temporal, left prefrontal–central, and left prefrontal–parietal regions. In the unpleasant-aroused condition, the prefrontal–temporal, prefrontal–parietal, and prefrontal–central regions showed the weakest connectivity.

**Figure 14:**
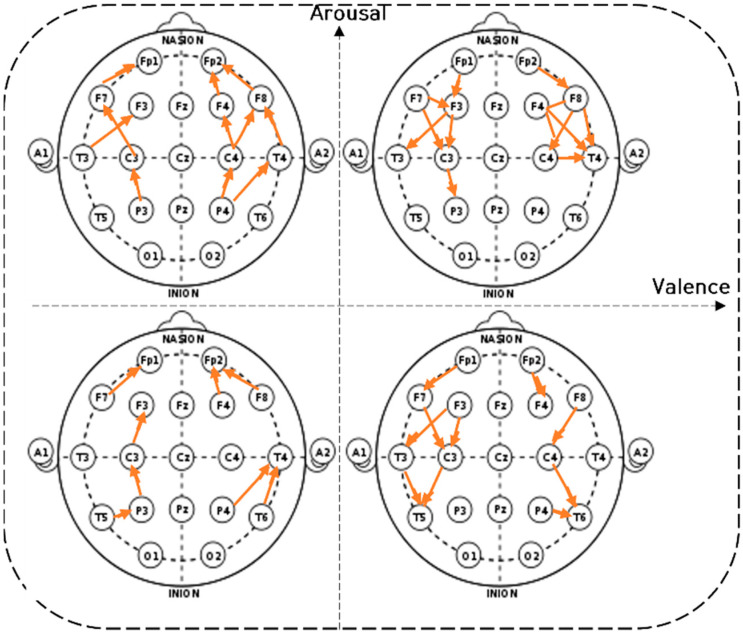
The short-distance connectivity of the prefrontal-temporal lobes, central-parietal lobes, and parietal-temporal lobes (SD connectivity) of the beta wave.

Figure 14 depicts the short-distance connectivity (SD connectivity) of the beta waves in the visual comparison diagram of the two-dimensional emotional model. In the aroused conditions (pleasant-aroused, unpleasant-aroused), strong frontal–temporal–central connectivity was observed. However, in the relaxed conditions (pleasant-relaxed, unpleasant-relaxed), strong central–parietal connectivity was observed.

In summary, the analysis suggests a strong frontal activity in the unpleasant-aroused condition, indicating intense information processing and transfer involving the frontal cortex. In pleasant conditions, feedback is sent to the parietal, temporal, and central regions after the prefrontal cortex processes the information. In the unpleasant-relaxed condition, brain connectivity implies the control of the participant’s eye movement.

#### 4.2.2. Power Value Analysis in Three Distance Connectivity

**Figure 15:**
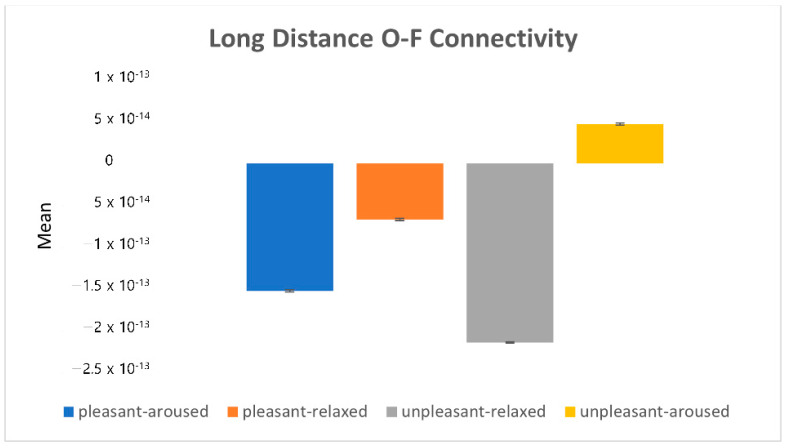
The eigenvalues in the long-distance O-F connectivity.

**Figure 16:**
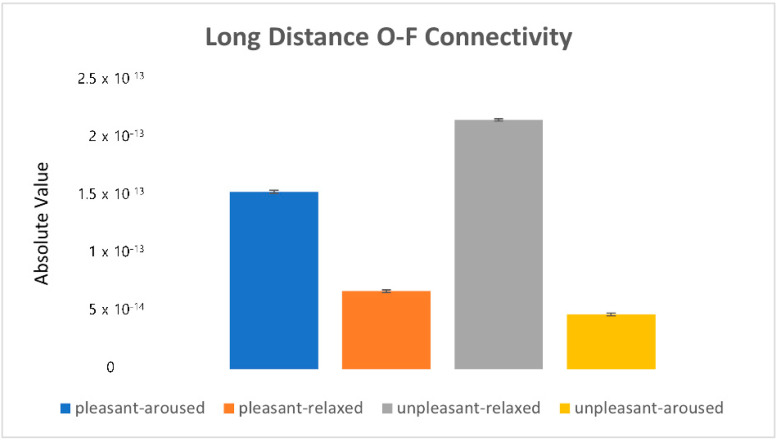
The absolute value in the long-distance O-F connectivity.

**Figure 17:**
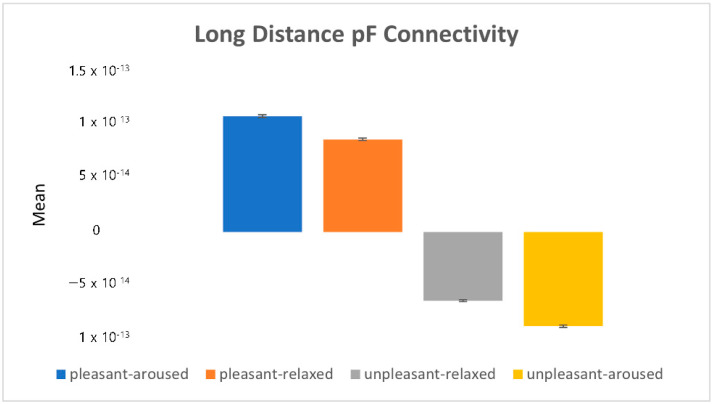
The eigenvalues in the long-distance prefrontal connectivity.

**Figure 18:**
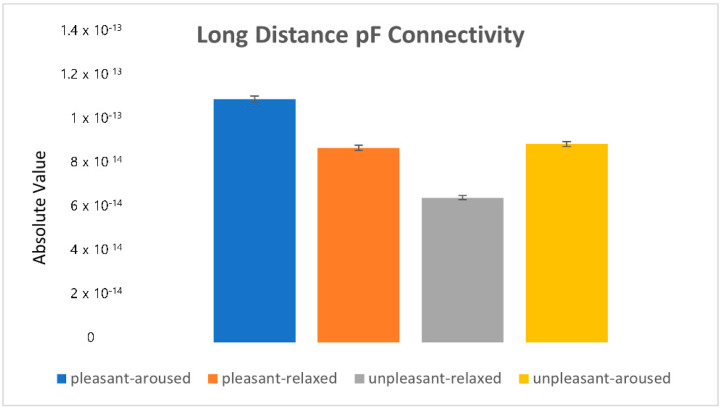
The absolute value in the long-distance prefrontal connectivity.

**Figure 19:**
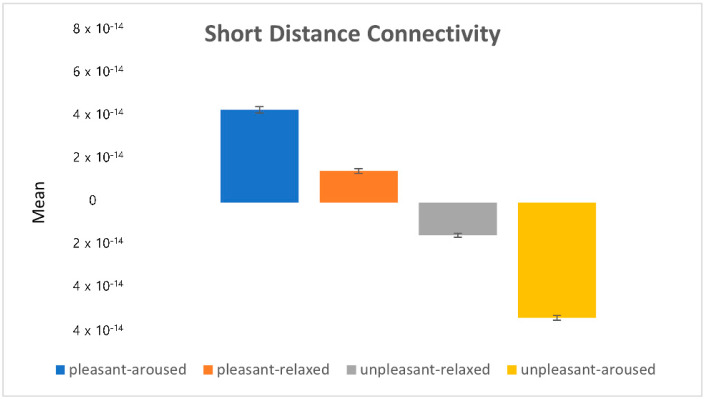
The eigenvalues in the short-distance connectivity.

**Figure 20:**
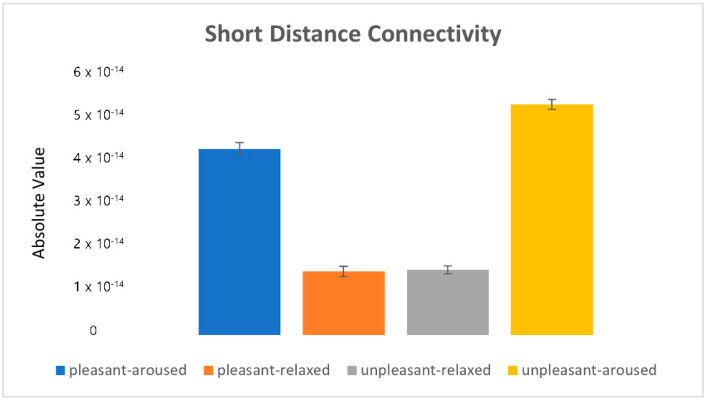
The absolute value in the short-distance connectivity.

To further understand the strength and directionality of brainwave connectivity, statistical analysis was performed on the power value using ANOVA, followed by post hoc analyses (see Figure 15, Figure 16, Figure 17, Figure 18, Figure 19 and Figure 20).

Figure 15 depicts the eigenvalues (i.e., mean power value) of the occipital and frontal lobe connectivity. The plus-minus sign of the eigenvalue determines the causality. In the unpleasant-aroused condition, more information is processed in the frontal lobe, indicating more activity in the occipital lobe than in primary visual processing.

Figure 16 shows the absolute values of the mean (|mean|). The pleasant-relaxed and unpleasant-aroused conditions exhibited high occipital-frontal connectivity, whereas the pleasant-relaxed condition exhibited left hemisphere-frontal activation (see Figure 12).

Figure 17 depicts the eigenvalues (i.e., the mean power value) of prefrontal connectivity. The plus-minus sign of the eigenvalue determines the causality. The results showed that activity in the prefrontal lobe in pleasant conditions (pleasant-aroused, pleasant-relaxed) was greater than that in other regions. Conversely, in the unpleasant conditions (unpleasant-aroused, unpleasant-relaxed), activity in the other regions was stronger than that in the prefrontal lobe.

Figure 18 shows the absolute values of the mean (|mean|). The unpleasant-relaxed condition exhibited the strongest connectivity.

Figure 19 depicts the eigenvalues (i.e., mean power value) of the short-distance connectivity in frontal–temporal, frontal–central, and temporal–parietal connections in the four emotion-eliciting conditions. Overall, connectivity in the relaxed condition was stronger than that in the aroused condition. Specifically, central–parietal connectivity showed stronger activity than frontal–temporal and frontal–central connectivity (see Figure 14).

Figure 20 shows the absolute values of mean (|mean|). The relaxed conditions (pleasant-relaxed and unpleasant-relaxed) showed stronger connectivity, specifically stronger P-O connectivity. Conversely, the aroused conditions (pleasant-aroused, unpleasant-aroused) showed weaker connectivity, but stronger F-T connectivity. In particular, the unpleasant-aroused, pleasant-aroused, and pleasant-relaxed conditions showed substantial premotor cortical PMDr (F7) connections associated with eye movement control. This was consistent with the saccade results.

Through statistical analysis, we found that connectivity in the pleasant-relaxed condition was the highest, while connectivity in the unpleasant-relaxed condition was higher than that in the pleasant-aroused and unpleasant-aroused conditions.

By comparing the three extracted brainwave connectivity eigenvalues with subjective evaluations, we found that the long-distance prefrontal connectivity eigenvalues have similar characteristics to the valence score measures of subjective evaluations. The prefrontal cortex (PFC) makes decisions and is responsible for cognitive control. Positive valence increases the neurotransmitter dopamine, enhancing cognitive control [28,29,30]. This may explain prefrontal activation in pleasant conditions (see Figure 15).

In summary, in the unpleasant-aroused condition, the frontal lobe showed a stronger activation than the occipital lobe. Overall, in pleasant conditions, the prefrontal lobe showed a stronger activation than other regions. Conversely, in unpleasant conditions, the prefrontal lobe showed a weaker activation than other regions.

### 4.3. Clustering Eye Movement Features

The statistical results showed that the short-distance connectivity eigenvalue and subjective evaluation arousal score had similar characteristics. Connectivity in the unpleasant-relaxed condition was the strongest (Figure 16). Specifically, central-parietal connectivity showed stronger connectivity than frontal–temporal and frontal–central connectivity. Unpleasant emotions are known to activate central–parietal connectivity [31].

**Figure 21:**
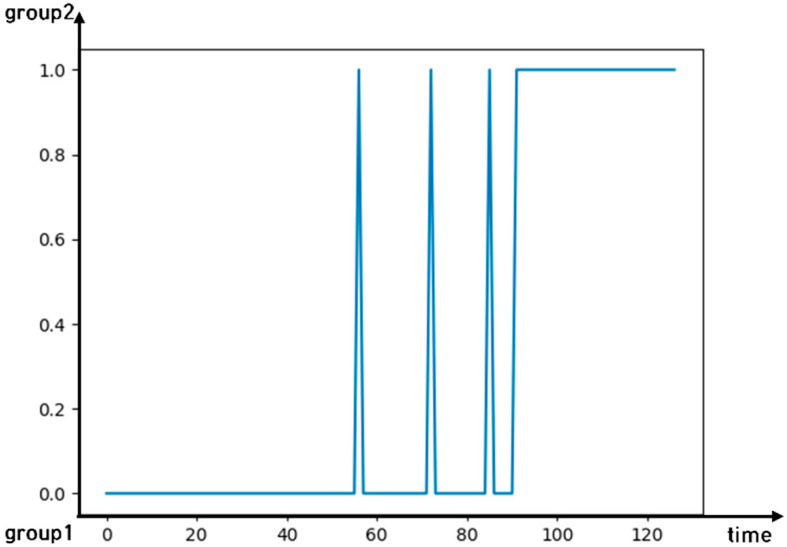
An instance of a participant’s k-Means results.

The three eigenvalues of the extracted EEG can be used to distinguish the four emotions in the two-dimensional emotional model. We conducted an unsupervised K-means analysis in chronological order using these three eigenvalues. We distinguished the emotional and non-emotional states of each participant while viewing the emotional video. The emotional and non-emotional states of the eye movement data were then distinguished. Figure 21 shows an instance of a participant’s K-means results. Group 1 indicates the non-emotional states, whereas Group 2 indicates the emotional states. The figure implies that the participant’s state changes from a non-emotional state (i.e., 0.0) to an emotional state (i.e., 1.0) as a function of time.

**Figure 22:**
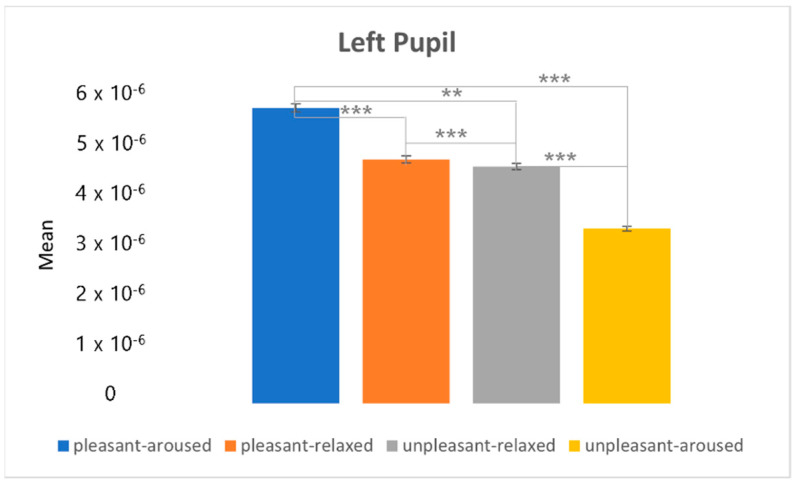
The post hoc analysis of the left pupil. ** *p* < 0.05. *** *p* < 0.001.

**Figure 23:**
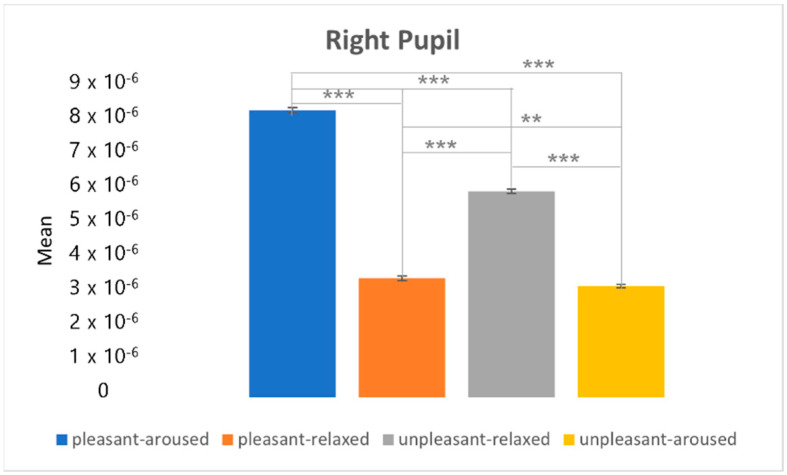
The post hoc analysis of the right pupil. ** *p* < 0.05. *** *p* < 0.001.

Figure 22 and Figure 23 depict the post-hoc analysis of the left and right pupils between the two-dimensional emotional model conditions. From the statistical results of the eye movement eigenvalues, the characteristics of the right pupil and left pupil did not change much between the four conditions; the pupil of the pleasant-aroused condition had the largest change, followed by the pleasant-relaxed and unpleasant-relaxed conditions. The least difference was observed in the unpleasant-aroused condition.

However, in relaxed conditions (pleasant-relaxed and unpleasant-relaxed), the right pupil of the unpleasant-relaxed condition was larger than the left pupil. From the first eigenvalue long-distance O-F connectivity of brain wave connectivity, we found two locations with high connectivity: the right occipital lobe and the left and right prefrontal lobes.

**Figure 24:**
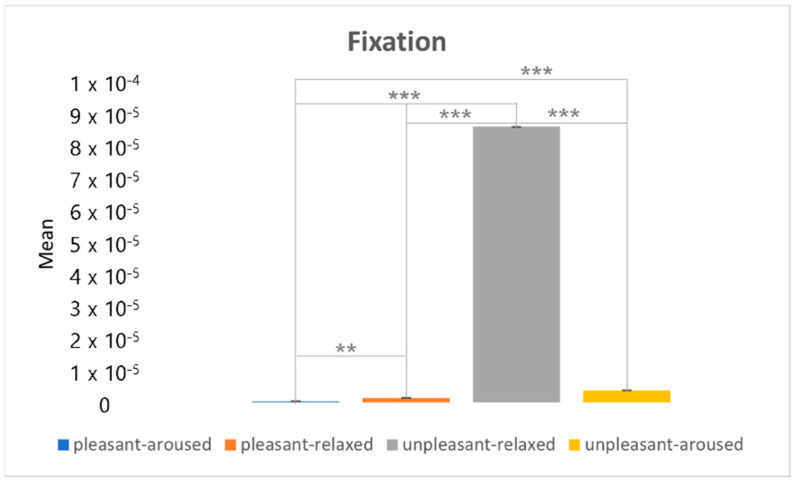
The post hoc analysis on the fixation. ** *p* < 0.05. *** *p* < 0.001.

Figure 24 shows the results of the post hoc analysis of the fixation between the two-dimensional emotional model conditions. The fixation feature in the unpleasant-relaxed condition was larger than that in the other three conditions.

**Figure 25:**
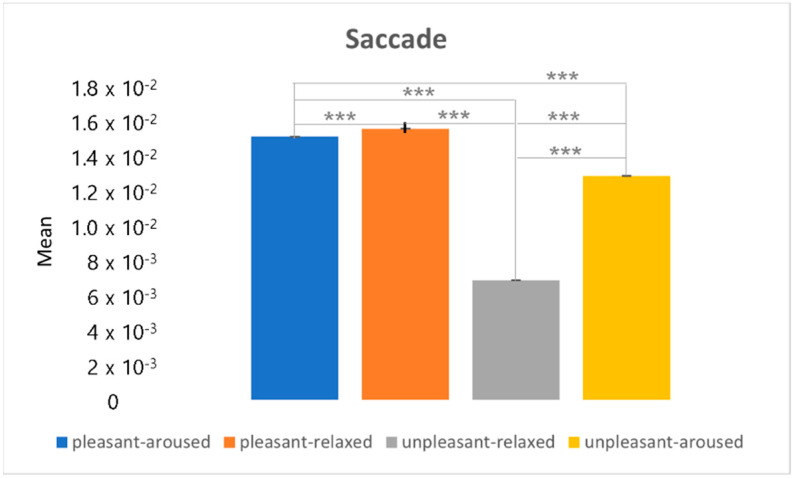
The post hoc analysis on the saccade. *** *p* < 0.001.

Figure 25 shows the results of the post hoc analysis of the saccade between the two-dimensional emotional model conditions. The results showed the lowest change in the unpleasant-relaxed condition, and the greatest change in the pleasant-relaxed condition. The characteristics of the saccades were similar to those of the short-distance connectivity eigenvalues. Short-distance connectivity also showed weak brain connections in the unpleasant-relaxed condition (see Figure 14). After the frontal lobe makes a cognitive judgment, it gives instructions to the occipital lobe, causing saccadic eye movements.

## 5. Conclusions and Discussion

This study aimed to understand the relationship between brain wave connectivity and eye movement characteristic values using a two-dimensional emotional model. We divided brainwave connectivity into three distinct groups: long-distance occipital–frontal connectivity, long-distance prefrontal connectivity between the prefrontal lobe and temporal lobe, parietal lobe, and central lobe, and short-distance connectivity including the characteristic relationships between the frontal lobe–temporal lobe, frontal lobe-central lobe, temporal–parietal lobe, and parietal lobe–central. Then, through unsupervised learning of these three eigenvalues, the emotional response was divided into emotional and non-emotional states in real time using K-means analysis. The two states were used to extract the feature values of the eye movements. We analyzed the relationship between eye movements and brain wave connectivity using statistical analyses.

The results revealed that the connectivity eigenvalues of the long-distance prefrontal lobe, temporal lobe, parietal lobe, and center are related to cognitive activity involving high valence. The prefrontal lobe occupies two-thirds of the human frontal cortex [32] and is responsible for recognition and decision-making, reflecting cognitive judgment from valence responses [33,34]. Specifically, the dorsolateral prefrontal cortex (dlPFC) is involved with working memory [35], decision making [36], and executive attention [37]. However, most recently, Nejati et al. [32] found that the role of dlPFC extends to the regulation of the valence of emotional experiences. Second, the saccade correlated with long-distance occipital-frontal connectivity. After making a judgment, the frontal lobe provides instructions to the occipital lobe, which moves the eye. Electrical stimulation of several areas of the cortex evokes saccadic eye movements. The prefrontal top-down control of visual appraisal and emotion-generation processes constitutes a mechanism of cognitive reappraisal in emotion regulation [38]. The short-distance connectivity results showed emotional fluctuations caused by the unconscious stimulation of audio-visual perception.

We acknowledge some limitations of the research. First, the results of our study are from one stimulus for each of the four quadrants in the two-dimensional model. Future studies may use multiple stimuli, possibly controlling the type of stimuli. Second, although pupillometry is an effective measurement for understanding brain activity changes related to arousal, attention, and salience [39], we did not find consistent and conclusive results between pupil size and brain connectivity. The size of pupils changes according to ambient light (i.e., pupillary light reflex) [40,41], which may have confounded the results. Future studies should control extraneous variables more thoroughly to find the main effect of pupil characteristics. Third, our analysis is based on participants of local university students, limiting the age range (i.e., 20 to 30 years). Age and culture may influence the results, so future studies may consider a broader range of demographic populations and conduct a cross-cultural investigation.

The study purposely analyzed brain connectivity and changes in eye movement in tandem to establish a relational basis between neural activity and eye movement features. We took the first step in unraveling such a relationship, albeit fell short in achieving a full understanding, such as the pupil size characteristics. Because the eyes’ structures are connected to the brain’s nerves, an exclusive analysis of eye features may lead to a comprehensive understanding of the participant’s emotions. A non-contact appraisal of emotion based on eye feature analysis may be a promising method applicable to metaverse or media art.

## Figures and Tables

**Table 1:** Comparison of previous and proposed methods.

Methods	Strengths	Weaknesses
Deep canonical correlation analysis (DCCA) of integrated functional features [22]	Applied machine learning and incorporated and analyzed brain connectivity and eye movement data.	The statistical significance of brain connectivity and eye movement feature variables was not analyzed.
Designed a six-electrode placement to collect EEG and combined them with eye movements to integrate internal cognitive states and external behaviors [24].	Demonstrated the effect of modality fusion with a multimodal deep neural network. The mean accuracy was 85.11% for four emotions (happy, sad, fear, and neutral).	The study did not analyze the functional relationship between brainwave connectivity and eye movements.
User-independent emotion recognition method to identify affective tags for videos using gaze distance, pupillary response, and EEG [25].	Investigated pupil diameter, gaze distance, eye blinking, and EEG and applied modality fusion strategy at both feature and decision levels.	The experimental session limited the number of videos shown to participants. The study did not investigate brainwave connectivity.
Recognition of emotion by brain connectivity and eye movement (proposed method).	Explored the characteristics of brainwave connectivity and eye movement eigenvalues and the relationship between the two in a two-dimensional emotional model.	Did not apply machine learning to formulate a model. The analysis was based on one stimulus for each of the four quadrants in the two-dimensional model.

**Table 2:** Multiple comparisons of mean arousal scores using Tukey HSD.

Emotion Condition 1	Emotion Condition 2	Mean Difference	Lower	Upper	Reject
Pleasant-aroused	Pleasant-relaxed	−2.2083	−2.8964	−1.5202	True
Pleasant-aroused	Unpleasant-aroused	0.9375	0.2494	1.6256	True
Pleasant-aroused	Unpleasant-relaxed	−0.7083	−1.3964	−0.0202	True
Pleasant-relaxed	Unpleasant-aroused	3.1458	2.4577	3.8339	True
Pleasant-relaxed	Unpleasant-relaxed	1.5	0.8119	2.1881	True
Unpleasant-aroused	Unpleasant-relaxed	−1.6458	−2.3339	−0.9577	True

**Table 3:** Multiple comparisons of mean valence scores using Tukey HSD.

Emotion Condition 1	Emotion Condition 2	Mean Difference	Lower	Upper	Reject
Pleasant-aroused	Pleasant-relaxed	−0.125	−0.6531	0.4031	False
Pleasant-aroused	Unpleasant-aroused	−3.625	−4.1531	−3.0969	True
Pleasant-aroused	Unpleasant-relaxed	−3.1042	−3.6322	−2.5761	True
Pleasant-relaxed	Unpleasant-aroused	−3.5	−4.0281	−2.9719	True
Pleasant-relaxed	Unpleasant-relaxed	−2.9792	−3.5072	−2.4511	True
Unpleasant-aroused	Unpleasant-relaxed	−1.6458	−2.3339	−0.9577	True
